# A comprehensive splicing characterization of COL4A5 mutations and prognostic significance in a single cohort with X-linked alport syndrome

**DOI:** 10.3389/fgene.2025.1564343

**Published:** 2025-06-11

**Authors:** Haomiao Li, Shengnan Zhang, Wei Zhou, Chunli Wang, Chunhua Zhu, Sanlong Zhao, Fei Zhao, Zhanjun Jia, Aihua Zhang, Bixia Zheng, Guixia Ding

**Affiliations:** ^1^ Department of Nephrology, Children’s Hospital of Nanjing Medical University, Nanjing, China; ^2^ Nanjing Key Laboratory of Pediatrics, Children’s Hospital of Nanjing Medical University, Nanjing, China

**Keywords:** alport syndrome, COL4A5, minigene assay, splicing, mRNA

## Abstract

**Introduction:**

X-linked Alport syndrome (XLAS), caused by mutations in the COL4A5 gene, is an X-linked hereditary disease typically characterized by renal failure, hearing loss, and ocular abnormalities. It is a leading hereditary cause of end-stage renal disease (ESRD) worldwide. Studies on the genotype-phenotype correlation in Alport syndrome suggest that splicing mutations result in more severe clinical phenotypes than missense mutations. Determining whether COL4A5 mutations lead to aberrant mRNA splicing is critical for diagnosis and prognosis.

**Methods:**

This study retrospectively reviewed pediatric XLAS patients with COL4A5 gene mutations from a single-center cohort, summarizing and analyzing their clinical features. Minigene assay was employed to evaluate the mRNA splicing functionality of 26 single-nucleotide variants (SNVs), both intronic and exonic, identified in XLAS patients. Bioinformatics tools were used to evaluate the accuracy and sensitivity of splicing mutation prediction. Additionally, linear mixed models were applied to analyze the relationship between mutation types and prognosis in patients’ estimated glomerular filtration rate (eGFR), exploring genotype-phenotype correlations.

**Results:**

In this cohort, we screened 41 XLAS pediatric patients, including 32 with confirmed XLAS and nine suspected XLAS. The cohort included 21 males (51.2%) and 20 females (48.8%), with a median age at onset of 4.42 years. Among the patients, 22 presented with both hematuria and proteinuria, while 18 exhibited hematuria alone. Notably, only one patient had isolated proteinuria. Regarding mRNA splicing, among the 26 intronic and exonic SNVs, 10 mutations (38.5%) were found to cause aberrant mRNA splicing, as demonstrated by the minigene assay. Sensitivity and specificity assessments of bioinformatics tools revealed that ESE Finder demonstrated higher sensitivity, while RNA Splicer exhibited greater specificity. Furthermore, These splicing abnormalities were closely associated with a faster decline in eGFR.

**Conclusion:**

This study demonstrates that 38.5% of SNVs in the COL4A5 gene result in aberrant mRNA splicing, which is closely linked to renal function decline in XLAS. Splicing mutations are correlated with more rapid renal progression, highlighting the importance of determining the splicing effects of SNVs during genetic screening for XLAS.

## 1 Introduction

Alport syndrome (AS) is a leading hereditary cause of end-stage renal disease (ESRD) worldwide, imposing a significant burden on patients and healthcare systems (Kashtan, 2021; Savige et al., 2022). The clinical features of AS include persistent hematuria, progressive proteinuria, and a gradual decline in renal function, often accompanied by sensorineural hearing loss and ocular abnormalities (Nozu et al., 2019; Savige et al., 2019). AS is caused by mutations in the COL4A3, COL4A4, and COL4A5 genes, which encode the α3, α4, and α5 chains of type IV collagen (Savige et al., 2022). These chains normally assemble into stable trimers that are crucial for maintaining the structure and filtration function of the glomerular basement membrane (GBM). Mutations in these genes disrupt the formation of these trimers, leading to structural defects in the α-chains, thickening, lamination, and splitting of the GBM, ultimately resulting in progressive renal dysfunction (Aoto et al., 2022; Savige et al., 2022; Boisson et al., 2023). In addition to kidney involvement, defective collagen affects basement membranes in the cochlea and eyes, contributing to extrarenal manifestations of AS (Hudson et al., 2003). AS can be classified based on inheritance patterns into X-linked Alport syndrome (XLAS), autosomal recessive Alport syndrome (ARAS), autosomal dominant Alport syndrome (ADAS), and digenic Alport syndrome. XLAS, caused by mutations in the COL4A5 gene, is the most common form, accounting for approximately 50% of AS cases (Morinière et al., 2014). Due to its X-linked pattern, male patients typically experience earlier onset and more rapid disease progression, often reaching ESRD by their second or third decade of life (Jais et al., 2000).

It has been well-documented that male patients with XLAS demonstrate a pronounced genotype-phenotype correlation, with splicing mutations notably associated with more severe clinical outcomes compared to missense mutations (Zeni et al., 2024). Advancements in high-throughput sequencing technologies have led to the identification of numerous COL4A5 mutations. However, determining their pathogenicity—particularly those affecting RNA splicing—remains challenging. Splicing abnormalities can result in exon skipping, cryptic splice site activation, or pseudoexon insertion, leading to nonsense-mediated mRNA decay or production of dysfunctional proteins (Groopman et al., 2019). Notably, while missense mutations are traditionally considered to alter protein function directly, they can also impact RNA splicing, thus contributing to splicing defects and more severe phenotypic outcomes. For instance, Aoto et al. (2022) reported that 85% of mutations at COL4A5 exon boundaries caused abnormal RNA splicing, with affected patients progressing to ESRD by age 27, compared to age 40 in those with missense mutations. Furthermore, Boisson et al. (2023) reported that 88% of XLAS patients missed by routine DNA sequencing exhibited splicing defects due to intronic mutations and exon single-nucleotide variants (SNVs), resulting in more severe kidney damage. Therefore, determining whether COL4A5 gene mutations result in aberrant mRNA splicing is crucial for the diagnosis and clinical prognosis of pediatric XLAS patients.

Bioinformatics tools are widely used to predict splicing defects but face limitations in specificity, often yielding high false-positive rates of up to 30%–40% (Ohno et al., 2018; Aoto et al., 2022). While these tools demonstrate high sensitivity (80%–90%) for canonical exon-intron boundary mutations, their accuracy drops significantly for deep intronic or low-frequency variants. Deep intronic mutation detection rates are approximately 30%–40%, compared to nearly 90% for common exon mutations. As these tools are typically trained on known datasets, their predictions for novel or rare mutations are less reliable (Ohno et al., 2018). Consequently, experimental validation is essential, particularly for complex splicing abnormalities.

In clinical practice, RNA from skin fibroblasts, hair follicle roots, or kidney tissue can be analyzed to detect splicing abnormalities in the COL4A5 gene (Wang et al., 2021). Additionally, urine-derived podocyte RNA offers a non-invasive, repeatable option for assessing kidney-specific splicing defects (Daga et al., 2018). However, urine-derived RNA quality is highly sensitive to storage conditions, increasing the risk of degradation and unreliable results. While RNA from biopsy samples provides valuable cell-specific insights, the invasive nature of these procedures limits their use, especially in pediatric patients (Daga et al., 2018). Furthermore, RNA sequencing struggles to detect low-abundance splicing events, particularly in heterogeneous tissues where abnormal transcript levels are minimal (Li and Dewey, 2011; Conesa et al., 2016). Minigene assay has emerged as a powerful tool for evaluating splicing defects. These assays circumvent the need for direct patient RNA samples, addressing issues of sample scarcity and preservation. They can be conducted in various cell lines, simulating tissue-specific splicing regulation, and have proven highly reliable in studies of genetic disorders involving splicing abnormalities (Putscher et al., 2021).

This study systematically evaluated the effects of exonic missense and intronic mutations in the COL4A5 gene on mRNA splicing by integrating bioinformatics predictions with minigene assays. Additionally, we aimed to determine whether these variants are splicing mutations to enhance the diagnosis and prognosis of AS patients. Out of 26 missense and intronic variants, 10 were reclassified as splicing mutations based on bioinformatics and functional assay results. Patients with splicing mutations exhibited a more pronounced decline in renal function compared to those with types of variants. This reclassification underscores the role of mRNA splicing in the pathogenic mechanisms of XLAS, thereby providing a robust foundation for clinical genetic diagnosis and the development of personalized treatment strategies.

## 2 Methods

### 2.1 Research patients and related definitions

This study retrospectively included 41 pediatric patients, comprising 32 with confirmed XLAS and 9 with suspected XLAS who carried COL4A5 gene mutations of uncertain pathogenicity, identified through genetic testing conducted at the nephrology outpatient clinic and inpatient ward of our hospital between 31 December 2017, and 31 December 2023. The data includes basic patient information (age, sex, age at initial diagnosis, and family history), genetic testing results, renal pathology biopsy findings, clinical manifestations (such as renal function, hearing loss, ocular changes, and other associated features), and information from the last follow-up.

The estimated glomerular filtration rate (eGFR) for pediatric patients in this study was calculated using the Schwartz formula: eGFR [ml·min^-1·^(1.73 m^2^)^−1^] = K × 88.4 × Height (cm)/Scr (μmol/L). The K value varies depending on the serum creatinine (Scr) measurement method and the patient’s age and sex. For Jaffe’s method, the value of K is set as follows: K = 0.33 for infants aged 0–28 days, K = 0.45 for children aged over 28 days to 1 year, K = 0.55 for children aged 2–12 years, and K = 0.77 for males and K = 0.55 for females aged over 12 years. For enzymatic methods, such as Creatinine Oxidase or Creatinine Imine Hydrolase, the value of K is 0.413 for all age groups. Renal dysfunction is defined as an eGFR less than 90 mL/min/1.73 m^2^, while ESRD is typically defined as an eGFR less than 15 mL/min/1.73 m^2^. Hearing loss was defined either by the presence of subjective symptoms or by pure tone audiometry findings (Olusanya et al., 2014). Ocular changes included maculopathy, lens irregularities, corneal opacities, cataracts, myopia, keratoconus, and astigmatism (Savige et al., 2015; De Silva et al., 2021).

### 2.2 Genetic testing

Genetic testing was performed by extracting genomic DNA using a DNA isolation kit (Tiangen, Beijing, China) in accordance with the manufacturer’s protocol. Genomic DNA was utilized for whole-exome sequencing (WES) and targeted next-generation sequencing (NGS), covering 148 associated genes detailed in Supplementary Table S1. The test was conducted on an Illumina HiSeq 2,500 platform, generating 150 bp paired-end reads. Sequencing data were aligned to the reference human genome (hg19 build), and insertions, deletions (INDELs), and SNVs were identified and filtered. Variants were annotated, and those with an allele frequency exceeding 1% were excluded based on databases such as dbSNP, an in-house MAF database, 1,000 Genomes MAF (Chinese), the Genome Aggregation Database (gnomAD), and ExAC. Each variant was annotated based on the NCBI reference sequence for COL4A5 (NM_000495). All variants were prioritized following ACMG (American College of Medical Genetics and Genomics) guidelines and confirmed through Sanger sequencing.

### 2.3 In silico analysis

Bioinformatics prediction of 26 COL4A5 gene SNVs at our center was performed using various tools. For pathogenicity analysis, Polymorphism Phenotyping v2 (PolyPhen_2) (http://genetics.bwh.harvard.edu/pph2/) and MutationTaster (https://www.mutationtaster.org/) were utilized. For RNA splicing prediction, the following tools were employed: EX-SKIP (https://ex-skip.img.cas.cz/), ESE Finder (https://esefinder.ahc.umn.edu/), SpliceAI (https://spliceailookup.broadinstitute.org/), BDGP (http://www.fruitfly.org/), and RNA Splicer (https://rddc.tsinghua-gd.org/tool/rna-splicer). We uesd Expasy (https://web.expasy.org/translate/) for protein translation prediction.

### 2.4 Construction of the plasmid

**FIGURE 1:**
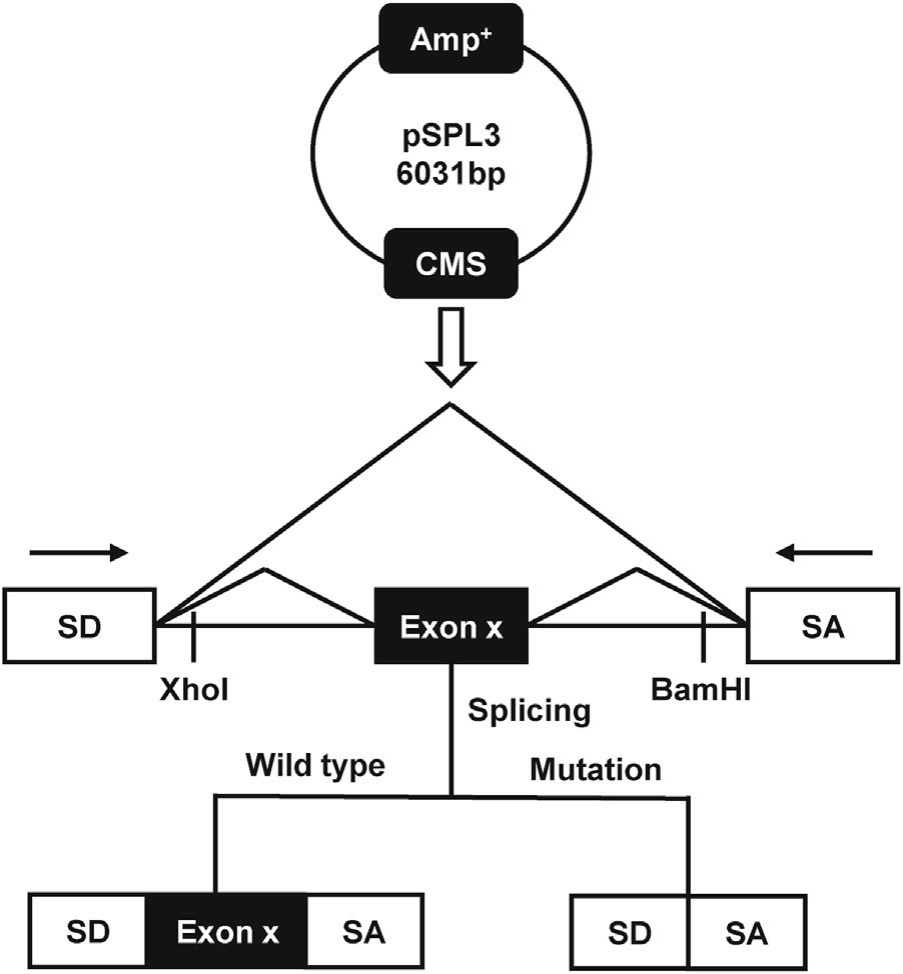
The schematic diagram of the minigene splicing assay constructed by pSPL3 exon trapping vector.

Genomic DNA from all wild-type and patient samples in our cohort was extracted from whole blood using a DNA isolation kit (Tiangen, Beijing, China), following the manufacturer’s guidelines. Primers were designed to amplify fragments containing a single exon, along with more than 100 base pairs of the upstream and downstream flanking intronic regions, incorporating XhoI and BamHI restriction sites. We designed primers (in Supplementary Table S2) using Primer-BLAST (https://www.ncbi.nlm.nih.gov/tools/primer-blast/) and amplified the sequences of COL4A5 using a polymerase chain reaction (PCR). Hybrid minigene constructs were generated using the pSPL3 vector, which includes a standard expression system with two exons (SD6 and SA2), to facilitate the analysis of the synthetic mRNA transcript (Zhou et al., 2020). The sequence of the upstream primer SD6 is 5′-TCT​GAG​TCA​CCT​GGA​CAA​CC-3′, and the downstream primer SA2 is 5′-ATC​TCA​GTG​GTA​TTT​GTG​AGC-3′. The PCR products were purified, sequenced, and subsequently cloned into the pSPL3 vector using XhoI and BamHI restriction enzymes, resulting in the construction of both wild-type and mutant minigene recombinants. The plasmid construction schematic is shown in Figure 1. The obtained wild-type and mutant plasmids were verified using single-direction sequencing.

### 2.5 Minigene assay

293 T cells were cultured in 12-well plates, using 1 mL of Dulbecco’s Modified Eagle Medium (10% fetal bovine serum). At 50%–70% confluence, 293 T cells were transfected with 1 μg of the recombinant plasmid using Lipofectamine 2000 (Invitrogen, Carlsbad, CA, USA) or PolyJet™ DNA Transfection Reagent (Signagen, Shanghai, China), following the manufacturers’ instructions. Total RNA was extracted from the cells 24 h after transient transfection using TRIzol reagent (Takara, Japan) and subsequently reverse-transcribed into cDNA using the PrimeScript™ RT Master Mix (Takara, Japan). PCR amplification was performed by the primers SD6 and SA2. The size and transcripts of the products of PCR were analyzed after agarose gel electrophoresis and sequenced. The marker used in the agarose gels was DL2000 Plus DNA Marker (Vazyme, China). The ratio of normal and abnormal transcripts was analyzed by grayscale value, and the experiment was repeated 3 times in each group. The software Image Lab was used to quantify grayscale value of each band. The following formula was used to quantify the percentage of aberrant splicing: exclusion percentage (%) = (Lowest band/(Lowest band + upper band)) × 100.

### 2.6 Statistical analysis

Statistical analysis was performed using SPSS 27.0 software. Normally distributed continuous data are presented as mean ± standard deviation (SD), and comparisons between two groups were made using the independent samples t-test. For non-normally distributed continuous data, the median (P25, P75) is reported, and comparisons between two groups were made using the Wilcoxon rank-sum test. Categorical data are presented as frequency (percentage), and comparisons between two groups were performed using the χ^2^ test or Fisher’s exact test. The exon exclusion percentage and intron retention percentage were analyzed with an unpaired Student’s t-test using GraphPad Prism (Version 8.0.1; GraphPad Software). Error bars represent the SEM (n = 3), and p < 0.05 was considered statistically significant.

Considering the variations in the number of observations and follow-up duration for each patient, the longitudinal changes in eGFR were analyzed using a linear mixed-effects model, which was fitted using the R package lmerTest (version 3.one to three, R.H. Bojesen Christensen). The model included patient identifiers (as random intercepts), age, mutation type, and their interaction with age, with eGFR as the dependent variable. Age, mutation type, and their interaction with age were treated as fixed effects, while patient identifiers were included as random intercepts. Statistical analysis was conducted using R version 4.4.1.

## 3 Results

### 3.1 Clinical characteristics

This study screened pediatric patients with XLAS (n = 32) and suspected XLAS (n = 9) at our center between 31 December 2017, and 31 December 2023, identifying 41 cases with COL4A5 gene mutations. Statistical analysis revealed that 21 patients (51.22%) were male and 20 (48.78%) were female. The age at onset was 4.42 (2.83, 7.83) years, while the age at the last follow-up was 6.79 ± 3.52 years. No significant differences were found between males and females in terms of age at onset (Z = −0.76, P = 0.45) or age at the last follow-up (Z = −1.58, P = 0.11). Family history of kidney disease was reported in 26 patients (in Supplementary Table S3; Supplementary Figure S1). Regarding renal manifestations, a total of 22 patients presented with both hematuria and proteinuria (63.64% male and 36.36% female). Additionally, 18 patients exhibited hematuria alone (38.89% male and 61.11% female), while only Patient seven had isolated proteinuria. 40 patients (97.56%) maintained eGFR >90 mL/min/1.73 m^2^ up to the last follow-up. A total of 29 patients underwent kidney biopsies, with 22 showing typical pathological changes consistent with Alport syndrome. Other findings included IgA deposition in 2 cases (Patient 1 and 26, in Table 1; Supplementary Table S3), mesangial proliferative glomerulonephritis (MsPGN) in 4 cases, focal segmental glomerulosclerosis (FSGS) in 1case (Patient 14, in Table 1; Supplementary Table S3) and minimal change disease (MCD) in 2 cases. Regarding extrarenal manifestations, one patient had sensorineural hearing loss, and six patients had ocular abnormalities (5 with refractive errors and 1 with retinal abnormalities). In terms of treatment, a total of 23 patients received renin-angiotensin-aldosterone system (RAAS) inhibitors. Among them, one out of five children with intronic mutations (20%) underwent RAAS inhibitor treatment. Of those with missense mutations, 11 out of 21 (52.38%) received treatment. Similarly, eight out of 11 (72.72%) patients with truncating mutations (including nonsense and frameshift mutations) were treated, while three out of four patients (75%) with CNVs also received RAAS inhibitors. Detailed data can be found in Tables 1 and Supplementary Table S3.

**TABLE 1:** Clinical characteristics of XLAS patients in our cohort.

Characteristics	Male (n = 21)	Female (n = 20)	Total (n = 41)
Onset age, years	5.71 ± 3.39	3.88 (2.87, 5.96)	4.42 (2.83, 7.83)
Age at last visit, years	7.75 ± 3.65	5 (3.4, 7.58)	6.79 ± 3.52
Family history	13 (61.9%)	13 (65%)	26 (63.41%)
Kidney phenotypes			
Hematuria	21 (100%)	19 (95%)	40 (97.56%)
Proteinuria	14 (66.67%)	9 (45%)	23 (56.1%)
eGFR≤90 mL/min/1.73 m^2^	2 (9.52%)	0 (0%)	2 (4.88%)
Serum creatinine, μmol/L			
at first visit	29.6 (23.35, 46)	28.65 ± 7.39	28 (23, 36)
at last visit	34.7 (26.75, 45.13)	31.63 ± 9.08	31 (26.25, 41.45)
eGFR, mL/min/1.73 m^2^			
at first visit	175.75 ± 57.7	184.8 ± 33.86	180.17 ± 47.23
at last visit	183.44 ± 57.07	178.77 ± 26.99	181.16 ± 44.49
Renal biopsy	n = 17	n = 12	n = 29
IgA deposition	1 (5.88%)	1 (5%)	2 (6.9%)
MsPGN	4 (23.53%)	0 (0%)	4 (13.79%)
FSGS	1 (5.88%)	0 (0%)	1 (3.45%)
MCD	0 (0%)	2 (10%)	2 (6.9%)
AS	13 (76.47%)	9 (45%)	22 (75.86%)
Extrarenal phenotypes			
Hearing loss	1 (4.76%)	0 (0%)	1 (2.44%)
Ocular changes	3 (14.29%)	3 (15%)	6 (14.63%)

Note: eGFR, estimated Glomerular Filtration Rate; MsPGN, mesangial proliferative glomerulonephritis; FSGS, focal segmental glomerulosclerosis; MCD, minimal change disease; AS, alport syndrome.

### 3.2 Genetic characteristics

**FIGURE 2:**
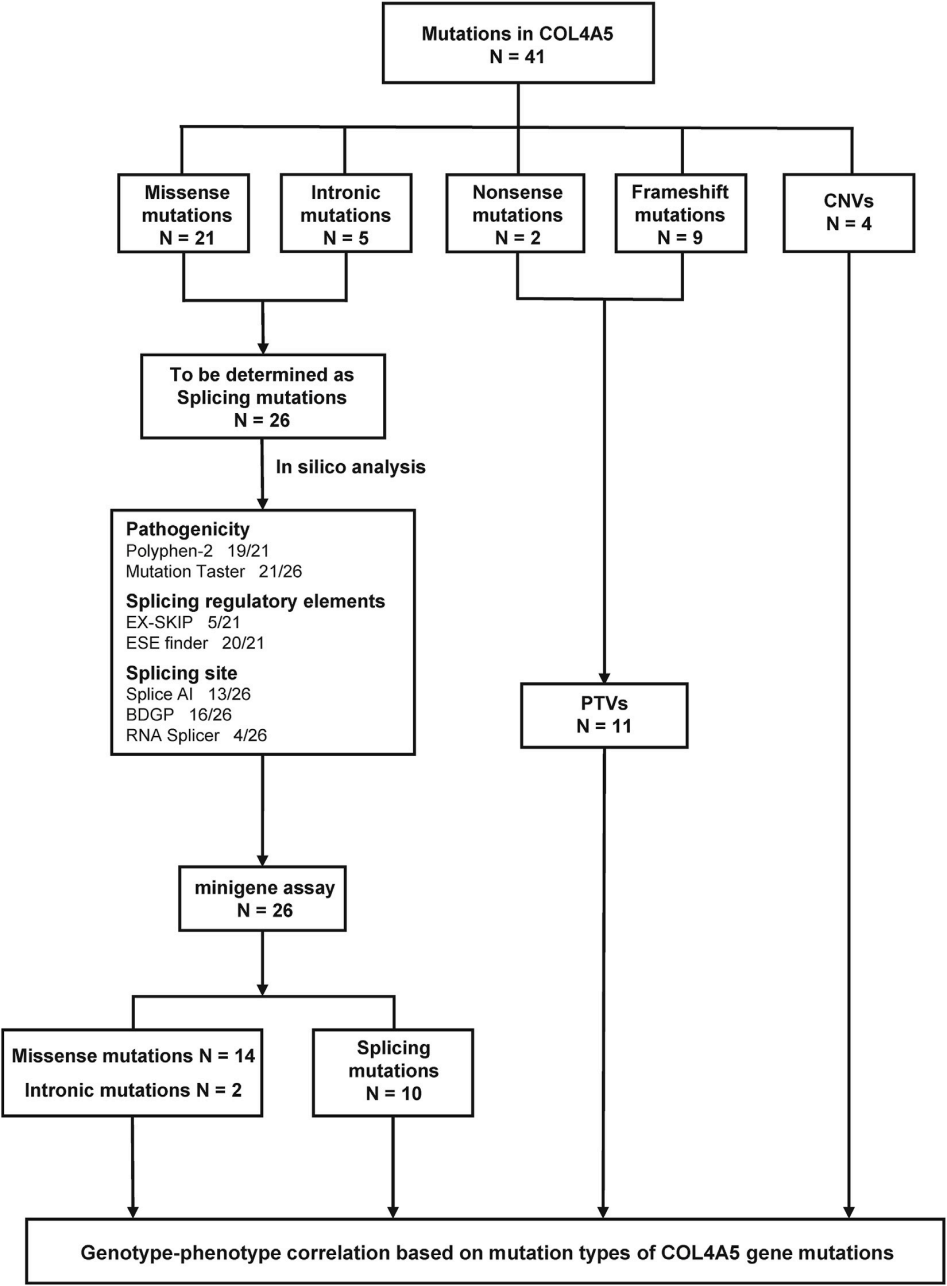
The flowchart of this study.

The mutations of COL4A5 gene in this cohort include 21 cases of missense mutations, 5 cases of intronic mutations, 2 cases of nonsense mutations, 9 cases of frameshift mutations, and 4 cases of copy number variation (CNV) (see Table 1). Among all patients, 26 mutations were novel, while 15 had been previously reported. According to the American College of Medical Genetics and Genomics (ACMG) guidelines (Richards et al., 2015), 17 mutations were classified as pathogenic, 14 as likely pathogenic, and 10 as of uncertain significance. Of the patients with mutations of uncertain significance, only three underwent kidney biopsy, and a definitive diagnosis of Alport syndrome (AS) was established based on characteristic biopsy findings in patient 27 (see Supplementary Table S3). All genetic mutation information and renal biopsy results are listed in Table 1 and Supplementary Table S3, and the flowchart is shown in Figure 2.

### 3.3 Aberrant splicing based on minigene assay

We conducted minigene assay on all 26 SNVs (see Supplementary Table S4), which included five intronic and 21 exonic variants. Overall, 10 (38.46%) SNVs (c.780 + 243 T>C, c.780 + 5G>A, c.4976 + 1G>A, c.638G>A, c.2329C>A, c.2605G>A, c.2624G>A, c.2678G>A, c.3319G>A and c.3667G>A) led to either complete or partial exon skipping. Although 5 SNVs (c.1634G>A, c.1781G>T, c.2228G>A, c.2314G>C and c.3059 T>C) caused partial exon skipping, the wild-type (WT) alleles for these children exhibited more exon skipping, and as such, they were not classified as splicing defects. In summary, we identified 10 SNVs that resulted in aberrant splicing, as detailed in Table 2.

**TABLE 2:** 10 SNVs lead to aberrant mRNA splicing.

Patient no.	Genotype information									
	Nucleotide	Intron/Exon	Exon length (bp)	Location in exon (bp)	Zygosity	Novel	Amino acid	ACMG classification	Evidence of pathogenicity	ACMG reClassification
1	c.780 + 243 T>C	intron13	_	_	het	Y	_	Uncertain	PS3*+PM2_Supporting	Likely pathogenic
2	c.780 + 5G>A	intron13	_	_	het	N	_	Uncertain	PS3*+PS4+PM2_Supporting	pathogenic
5	c.4976 + 1G>A	intron50	_	_	hemi	Y	_	Pathogenic	PVS1+PS2+PS3* +PM2+PM + PP1	Pathogenic
8	c.638G>A	exon11	36	+8	hemi	Y	p.G213E	Pathogenic	PS3*+PS4+PM1+ PM2+PM5+PP3	Pathogenic
18	c.2329C>A	exon29	151	+67	het	Y	p.R777S	Uncertain	PS3*+PM1+PM2_Supporting	Likely pathogenic
19	c.2605G>A	exon31	168	+73	hemi	N	p.G869R	Pathogenic	PS1+PS3*+PM1+ PM2+PP3+PP4	Pathogenic
20	c.2624G>A	exon31	168	+54	het	N	p.G875E	Likely pathogenic	PS1+PS3*+PM2+PP3	Pathogenic
21	c.2678G>A	exon32	90	−1	het	Y	p.G893D	Likely pathogenic	PS3*+PM1+PM2+ PM5+PP3+PP4	Pathogenic
23	c.3319G>A	exon37	127	+55	het	Y	p.G1107R	Pathogenic	PS1+PS3*+PS4+PM1+ PM2_Supporting + PP3_Strong	Pathogenic
24	c.3667G>A	exon41	186	−63	hemi	Y	p.G1223S	Likely pathogenic	PS3*+PM1+PM2_Supporting+ PM5+PP3_Strong	Pathogenic

In silico prediction																			Minigene assay	Effect on protein	a5(IV) staining in GBM
Polyphen-2	Mutation taster	EX-SKIP	ESE finder											Splice AI			BDGP	RNA splicer			
			WT						MUT					Δ type		Δscore					
			SF2/ASF		SF2/ASF	SC35	SRp40	SRp55	SF2/ASF	SF2/ASF	SC35	SRp40	SRp55								
_	P	_	_		_	_	_	_	_	_	_	_	_	_		0	NC	NC	Exon13 skipping	p. G230_Q260del31	NA
_	DC	_	_		_	_	_	_	_	_	_	_	_	Donor Loss		0.36	5′DS:1→0.96 (4%)	NC	Exon13 skipping	p. G230_Q260del31	NA
_	DC	_	_		_	_	_	_	_	_	_	_	_	Donor Loss		0.77	5′DS:0.92→NA	Inserting 189 bp/Deleting 173bp	Exon50 skipping	p.M1601*	Segmental
PD	DC	WT	0		1	2	2	0	0	1	1	2	0	Acceptor Gain/Donor Gain		0.01	3′AS:0.55→0.56 (2%)	NC	Exon11 skipping	p. G204_K215del12	Segmental
B	P	MUT	0		0	0	0	0	0	0	0	1	0	Donor Loss		0.24	5′DS:0.63→0.67 (6%)	NC	Exon29 skipping	p.M1delinsM810	NA
PD	DC	Both	3		3	1	0	0	2	4	2	1	0	_		0	3′AS:0.70→0.71 (1%)	NC	Exon31 skipping	p. G837_P892del56	Absence
PD	DC	WT	0		1	2	2	0	0	1	2	1	0	_		0	3′AS:0.70→0.41 (41%)	NC	Exon31 skipping	p. G837_P892del56	NA
PD	DC	Both	1		1	1	2	0	1	1	1	1	0	Acceptor Loss		0.44	5′DS:0.79→NA, 3′AS:0.79→0.47 (40%)	NC	Exon32 skipping	p. T894_G923del30	Absence
PD	DC	MUT	0		1	1	1	0	0	1	1	1	0	_		0	NC	NC	Exon37 skipping	p. K1083Nfs*10	Segmental
PD	DC	WT	1		2	2	1	0	0	1	2	0	0	_		0	3′AS:0.89→0.88 (1%)	NC	Exon41 skipping	p. Q1203_G1264del62	Absence

Note:Y: yes; N: no; ACMG, american college of medical genetics and genomics; PD, probably damaging; B, benign; DC, disease causing; P, polymorphism; WT, wild type; MUT, mutation; Both, Both the wild-type and mutation can exhibit exon skipping; NC, no changes; NA, not available; _: Absent.

“+” indicates distance from the 5′end of the exon and “−” represents distance from the 3′end.

### 3.4 Intronic SNVs affecting mRNA splicing

**FIGURE 3:**
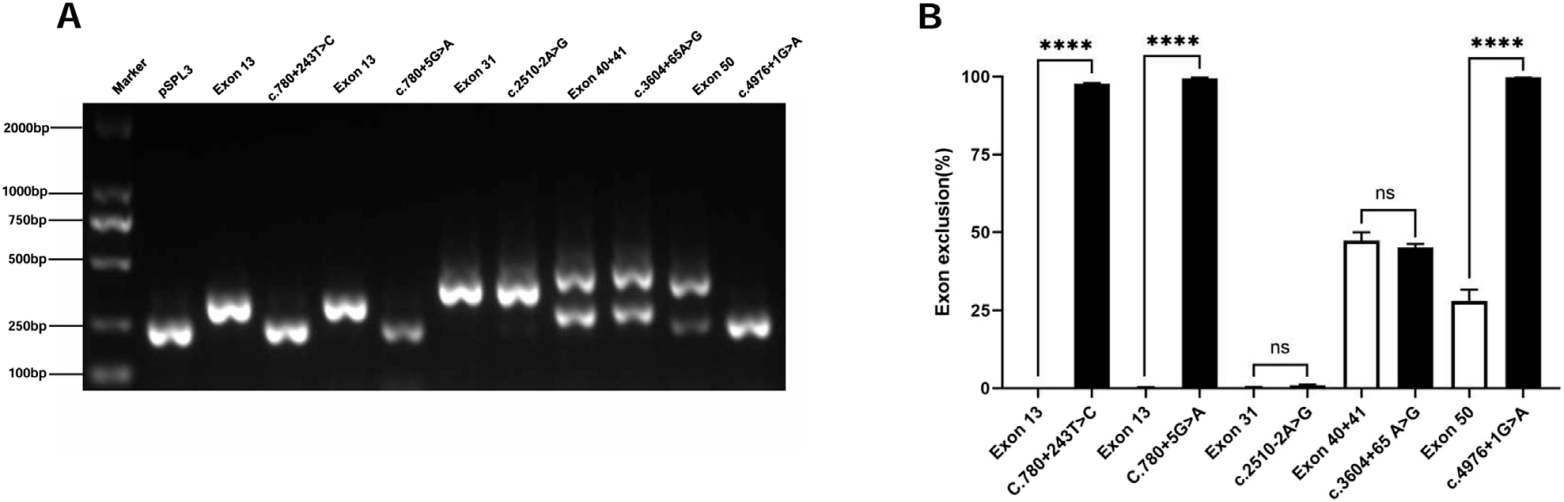
Agarose gel electrophoresis and statistical analysis of RT-PCR products of Intronic SNVs in the COL4A5 gene. Note **(A)** Lane 1: Marker DL 2000; Lane 2: pSPL3 (263 bp); Lane 3: Exon 13 (356 bp); Lane 4: c.780 + 243 T>C (263 bp); Lane 5: Exon 13 (356 bp); Lane 6: c.780 + 5G>A (263 bp); Lane 7: Exon 31 (431 bp); Lane 8: c.2510–2A>G (431 bp); Lane 9: Exon 40 + 41 (263 bp and 500 bp); Lane 10: c.3604 + 65A>G (263 bp and 500 bp); Lane 11: Exon 50 (263 bp and 436 bp); Lane 12: c.4976 + 1G>A (263 bp). **(B)** Quantification is expressed as the percentage (%) of exon skipping; *p < 0.05; **p < 0.01; ***p < 0.001; ****p < 0.0001.

This study included five intronic SNVs and complemented the minigene-based *in vitro* splicing assay, as shown in Figure 3. Minigene assay revealed that the classic splice mutation c.4976 + 1G>A resulted in partial skipping of exon 50 compared to the wild-type. The deep intronic mutations c.780 + 243 T>C and c.780 + 5G>A caused complete skipping of exon 13. However, the classic splicing site mutation c.2510–2A>G and the deep intronic mutation c.3604 + 65A>G did not result in any aberrant splicing. Prior to minigene assay, the pathogenicity of the deep intronic mutations c.780 + 243 T>C, c.780 + 5G>A, and c.3604 + 65A>G remained unclear. The minigene assay results reclassified c.780 + 243 T>C as likely pathogenic and c.780 + 5G>A as pathogenic, with strong support from additional evidence PS3 in accordance with ACMG guidelines. Regarding renal biopsy, the c.3604 + 65A>G mutation, corresponding to patient four (in Supplementary Table S1), showed normal α5 immunofluorescence in kidney biopsy, whereas the c.4976 + 1G>A mutation, corresponding to patient five (in Supplementary Table S1), demonstrated segmental loss of α5 immunofluorescence in kidney biopsy.

### 3.5 Exonic SNVs affecting mRNA splicing

**FIGURE 4:**
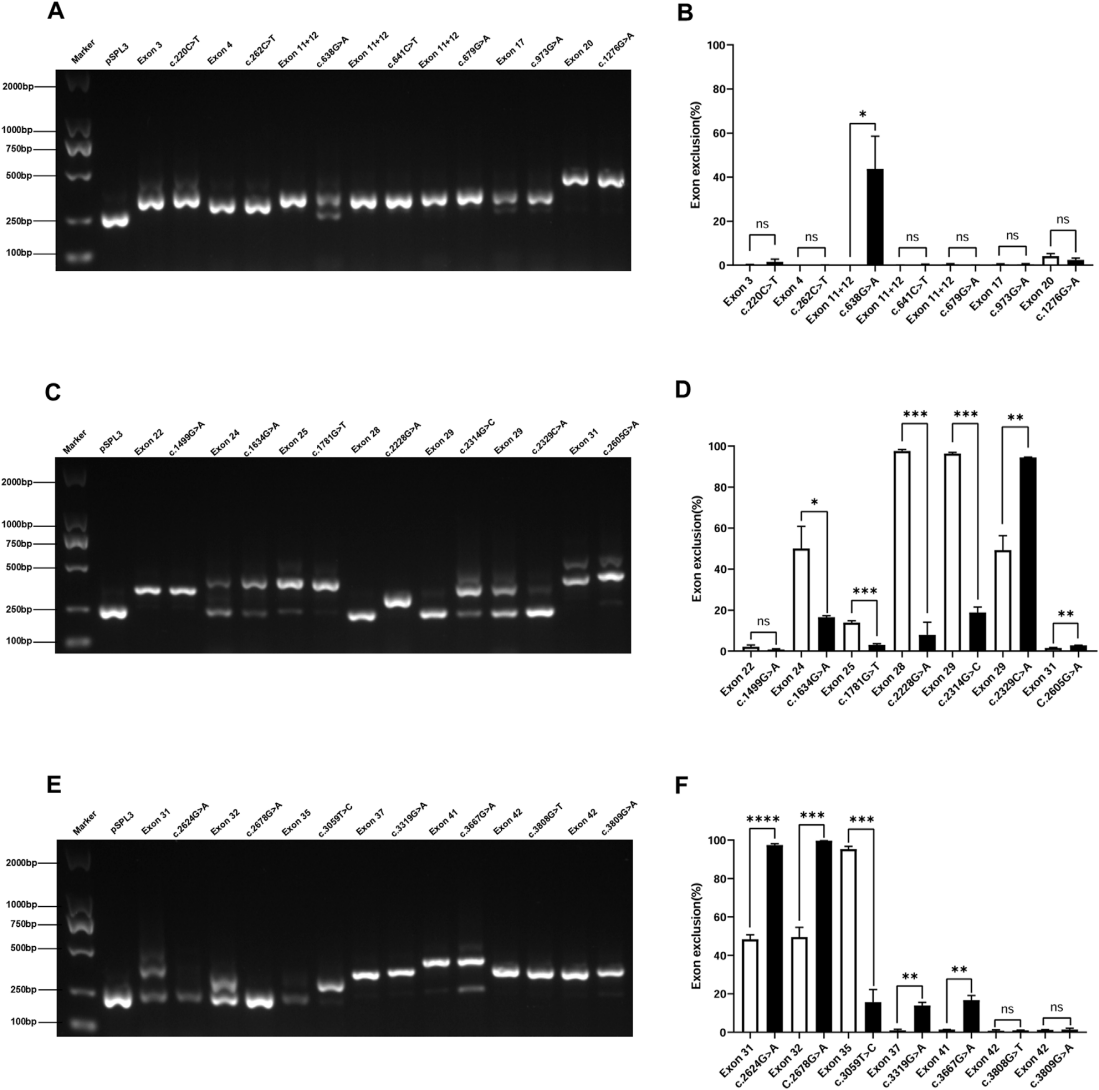
Agarose gel electrophoresis and statistical analysis of RT-PCR products of exonic SNVs in the COL4A5 gene. Note **(A)** Lane 1: Marker DL 2000; Lane 2: pSPL3 (263 bp); Lane 3: Exon 3 (353 bp); Lane 4: c.220C>T (353 bp); Lane 5: Exon 4 (308 bp); Lane 6: c.262C>T (308 bp); Lanes 7, 9, 11: Exon 11 + 12 (341 bp); Lane 8: c.638G>A (263 bp and 341 bp); Lane 10: c.641C>T (341 bp); Lane 12: c.679G>A (341 bp); Lane 13: Exon 17 (317 bp); Lane 14: c.973G>A (317 bp); Lane 15: Exon 20 (437 bp); Lane 16: c.1276G>A (437 bp) **(C)** Lane 1: Marker DL 2000; Lane 2: pSPL3 (263 bp); Lane 3: Exon 22 (356 bp); Lane 4: c.1499G>A (356 bp); Lane 5: Exon 24 (263 bp); Lane 6: c.1634G>A (263 bp and 455 bp); Lane 7: Exon 25 (432 bp); Lane 8: c.1781G>T (432 bp); Lane 9: Exon 28 (263 bp); Lane 10: c.2228G>A (361 bp); Lane 11: Exon 29 (263 bp); Lane 12: c.2314G>C (263 bp and 414 bp); Lane 13: Exon 29 (263 bp and 414 bp); Lane 14: c.2329C>A (263 bp); Lane 15: Exon 31 (431 bp); Lane 16: c.2605G>A (431 bp). **(E)** Lane 1: Marker DL 2000; Lane 2: pSPL3 (263 bp); Lane 3: Exon 31 (263 bp and 431 bp); Lane 4: c.2624G>A (263 bp); Lane 5: Exon 32 (263 bp and 353 bp); Lane 6: c.2678G>A (263 bp); Lane 7: Exon 35 (263 bp); Lane 8: c.3059T>C (353 bp); Lane 9: Exon 37 (390 bp); Lane 10: c.3319G>A (390 bp); Lane 11: Exon 41 (449 bp); Lane 12: c.3667G>A (263 bp and 449 bp); Lanes 13, 15: Exon 42 (397 bp); Lane 14: c.3808G>T (397 bp); Lane 16: c3809G>A (397 bp) **(B, D and F)** Quantification is expressed as the percentage (%) of exon skipping; *p < 0.05; **p < 0.01; ***p < 0.001; ****p < 0.0001.

This study included 21 exonic SNVs, and minigene-based *in vitro* splicing assays were performed for all of them, as shown in Figure 4. Minigene assay revealed that compared to the wild-type, the mutations c.638G>A, c.2329C>A, c.2605G>A, c.3319G>A, and c.3667G>A caused partial exon skipping; c.2624G>A and c.2678G>A resulted in complete exon skipping. Based on minigene assay, the remaining exonic SNVs did not induce mRNA splicing abnormalities. Among the 21 exonic SNVs, the pathogenicity of mutations c.220C>T, c.641C>T, c.2228G>A, c.2329C>A, and c.3059 T>C remained unclear prior to the minigene assay. Following the minigene assay and in accordance with ACMG guidelines, the mutation c.2329C>A was reclassified as likely pathogenic, and three variants (c.2624G>A, c.2678G>A, and c.3667G>A), initially classified as likely pathogenic, were reclassified as pathogenic, all supported by the additional evidence PS3. Regarding renal biopsy, α5 immunofluorescence was segmentally absent in cases of c.638G>A and c.3319G>A, and completely absent in cases of c.2605G>A, c.2678G>A, and c.3667G>A. In children with complete renal biopsy, SNVs causing partial or complete exon skipping were consistently associated with the loss of α5 immunofluorescence.

### 3.6 In silico analysis

All mutations were subjected to bioinformatic predictions regarding pathogenicity and splicing functionality, as summarized in Supplementary Table S4. The pathogenicity prediction results of Polyphen-2 and Mutation Taster for exonic SNVs are consistent, with 3 SNVs classified as benign (c.220C>T, c.2329C>A and c.3059 T>C), while the remaining 23 SNVs are predicted to be potentially pathogenic. According to Mutation Taster’s predictions, for intronic SNVs, 3 SNVs are potentially pathogenic (c.780 + 5G>A, c.2510–2A>G and c.4976 + 1G>A), while 2 SNVs are benign (c.780 + 243 T>C and c.3604 + 65 A>G). However, after further minigene assay, the benign SNVs (c.780 + 243 T>C and c.2329C>A) predicted by the algorithms both exhibited exon skipping, which may lead to the production of truncated proteins, a finding inconsistent with the original predictions.

To further clarify the sensitivity and specificity of the splicing prediction tools, we performed the corresponding calculations based on the results of the minigene assay in this study. Regarding the splicing regulatory elements, the sensitivity/specificity of the EX-SKIP and ESE Finder was 28.57%/78.57%, 100%/7.14%, respectively. For splicing site predictions, the sensitivity/specificity of the Splice AI, BDPG and RNA Splicer was 50%/50%, 80%/50%, 10%/81.25%, respectively.

### 3.7 Genotype–phenotype correlation

Based on the minigene assay results, we reclassified the patients in this cohort into four groups: missense mutations, splicing mutations, truncating mutations, and CNVs. Due to the small sample size, both male and female patients were included in the genotype-phenotype correlation study. Even though both genders were included, there were only 4 cases of CNVs, which could affect the stability of the linear mixed model; therefore, CNVs analysis was not performed. Further studies with more CNV cases are needed for validation.

**FIGURE 5:**
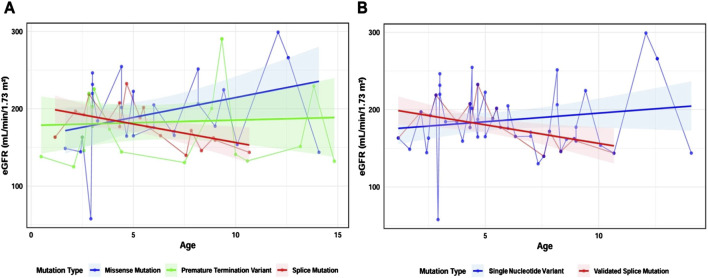
Constructing a linear mixed model based on mutation types. Note **(A)** Linear mixed model of three mutation types: missense mutation group (blue, n = 14), splicing mutation group (red, n = 10), and truncating mutation group (green, n = 11) **(B)** Linear mixed model of SNVs and splicing mutations after minigene analysis: SNV group (blue, n = 26) and splicing mutation group (red, n = 10). Shaded areas represent the 95% confidence interval (95% CI). Dots indicate eGFR values at different ages for individual patients. Straight lines connect data points from the same individual.

We used a linear mixed model with eGFR as the dependent variable, and age, mutation type, and their interaction with age as fixed effects, while patient identifiers were included as random intercepts. Linear models for missense mutations, splicing mutations, and truncating mutations were constructed (Figure 5A), and there was no statistically significant difference in the proportion of males across these three groups (χ^2^ = 0.75, p = 0.69). In the missense mutation group (n = 14), the model slope was 5.13 mL/min/1.73 m^2^/year [95% CI: 0.65 to 9.61]; in the splicing mutation group (n = 10), the model slope was −4.8 mL/min/1.73 m^2^/year [95% CI: −17.53 to 7.93]; and in the truncating mutation group (n = 11), the model slope was 0.69 mL/min/1.73 m^2^/year [95% CI: −9.93 to 11.31].

To investigate the impact of SNVs causing mRNA splicing defects on clinical phenotypes, we included the 26 intronic and exonic SNVs in the SNV group, and the 10 SNVs confirmed by minigene assay to cause abnormal mRNA splicing in the validated splicing mutation group. We constructed linear mixed models using the same approach. There was no statistically significant difference in the proportion of males between the two groups (χ^2^ = 0.03, p = 0.87). Notably, the slope of the SNV group was 2.23 mL/min/1.73 m^2^/year [95% CI: −0.94 to 5.4], while the slope for the validated splicing mutation group was −4.8 mL/min/1.73 m^2^/year [95% CI: −17.53 to 7.93], and the difference between these two groups was statistically significant (t = −2.03, P = 0.046), suggesting that splicing mutations are associated with a faster decline in eGFR (Figure 5B).

## 4 Discussion

In this study, we used minigene assay to validate whether 26 intronic and exonic SNVs from our cohort affect mRNA splicing. We found that 10 of these SNVs resulted in aberrant mRNA splicing. Furthermore, we correlated mRNA splicing abnormalities with clinical phenotypes and observed that patients with aberrant splicing experienced a more rapid decline in eGFR compared to those with normal splicing. This suggests that mRNA splicing abnormalities may serve as important indicators for predicting disease severity and progression. Additionally, based on the results of the minigene assay, we assessed the sensitivity and specificity of the corresponding bioinformatics prediction tools, providing valuable guidance for clinical decision-making.

Pre-mRNA splicing, a critical step in eukaryotic gene expression, involves the precise removal of non-coding regions (introns) and the joining of coding regions (exons) to produce mature mRNA. This process is mediated by the spliceosome, a ribonucleoprotein complex that ensures accurate recognition of exon-intron boundaries in pre-mRNA (Wahl et al., 2009). The exon-intron boundaries in pre-mRNA contain highly conserved sequences that act as splice recognition signals. The 5′and 3′ends of introns feature GT-AG canonical splice sites, known as the donor (5′SS) and acceptor (3′SS) sites, which are “cis-acting” elements recognized by small nuclear RNAs (snRNAs). Additional conserved sequences, such as the branch point (BP) and polypyrimidine tract (PPT), are also critical for facilitating interactions between the spliceosome and pre-mRNA. Beyond these canonical signals, regulatory elements, including exon splicing enhancers (ESE), exon splicing silencers (ESS), intron splicing enhancers (ISE), and intron splicing silencers (ISS), influence spliceosome selectivity by recruiting trans-acting factors (Canson et al., 2020). Splice mutations can arise not only at canonical splice sites but also at non-canonical regions, such as single-nucleotide substitutions within introns or exons. These mutations may lead to exon skipping, loss of exonic sequences, or aberrant inclusion of intronic or pseudoexonic regions (Boisson et al., 2023). In this study, we analyzed 21 cases of missense mutations, eight of which (38.1%) resulted in aberrant mRNA splicing. Previous research (Aoto et al., 2022) has shown that single-nucleotide substitutions at the last nucleotide of COL4A5 exons frequently disrupt mRNA splicing. However, the impact of substitutions at other exonic positions and their positional characteristics remain unclear. In diseases like amyotrophic lateral sclerosis and autosomal dominant polycystic kidney disease, certain “hot spot exons” are particularly susceptible to splice-disrupting mutations and exon skipping (Glidden et al., 2021). These exons often harbor clustered binding sites for RNA-binding proteins, which regulate splicing by recognizing specific RNA motifs (Fu and Ares, 2014). In our cohort, only the missense mutation c.2678G>A at the last nucleotide of exon 32 caused aberrant mRNA splicing, with no consistent pattern observed for other exonic mutations. This finding highlights the importance of mRNA analysis in XLAS patients with rapid disease progression to identify potential splice site mutations.

In terms of bioinformatics prediction, based on the results of the minigene assay, we found that the ESE Finder exhibited high sensitivity, while RNA Splicer showed high specificity. Different bioinformatics prediction tools exhibit varying levels of sensitivity and specificity. Therefore, relying solely on a single bioinformatics tool may not be sufficient to detect splicing-related variants (Aoto et al., 2022). It is advisable to use multiple bioinformatics prediction tools in parallel, and to further validate predictions through *in vitro* and *in vivo* experiments to confirm whether there are any abnormalities in mRNA splicing functionality.

Studies on the genotype-phenotype correlation in XLAS have shown that patients with splicing mutations exhibit more severe renal prognosis compared to those with missense mutations (Kamura et al., 2020; Yamamura et al., 2020b). In European populations, males with large deletions, nonsense mutations, or frame-shifting mutations in the COL4A5 gene face a 90% risk of developing ESRD by age 30, compared to 70% and 50% for those with splicing and missense mutations, respectively (Jais et al., 2000). In the Chinese population, the median age of ESRD onset in XLAS males with non-truncating and truncating mutations in the COL4A5 gene is 39 years and 22 years, respectively (Di et al., 2022). In our study, the renal biopsy results from patients with aberrant mRNA splicing showed the absence of the α5 chain, suggesting that aberrant mRNA splicing may affect the corresponding protein expression *in vivo*. Regarding clinical phenotypes, we only analyzed the impact of missense mutations, splicing mutations, and truncating mutations on eGFR, as there were fewer patients with CNVs. From the slope of the model, we observed that the splicing mutation group showed the most rapid decline in renal function, rather than the truncating mutation group. We hypothesize the following reasons: First, based on the treatment data of the patients, we found that a higher proportion of patients with truncating mutations (72.72%) were treated with RAAS inhibitors, compared to 60% of those with verified splicing mutations. Second, since Alport syndrome typically manifests renal dysfunction between the ages of 20 and 40, only one patient in our cohort had renal dysfunction, necessitating further follow-up to obtain more accurate genotype-phenotype correlations (Di et al., 2022). However, when comparing the SNV group and the group with SNVs leading to splicing mutations, we found that splicing mutations had a more significant impact on renal function, which is consistent with previous studies. This suggests that our early diagnosis and treatment of splicing mutations have been insufficient, highlighting the need for enhanced screening of splicing mutations to support diagnosis and treatment.

In this study, based on minigene assay of 26 COL4A5 gene SNV mutations, we found that 38.5% of these SNVs resulted in aberrant mRNA splicing. This finding provides epidemiological evidence for the role of SNVs in causing mRNA splicing abnormalities in XLAS, highlighting that such mutations significantly impact mRNA splicing. Although the sample size is relatively small, these results strongly support the potential effect of non-canonical splice sites and suggest that clinical diagnostics should pay particular attention to whether SNVs may lead to splicing defects. Given that this is a single-center study, future research should validate these findings with multi-center data and a larger sample size to draw more comprehensive conclusions. By doing so, we can gain deeper insights into the impact of aberrant mRNA splicing on the clinical manifestations of Alport syndrome, which may ultimately lead to improved diagnostic and therapeutic strategies.

Several gene therapy strategies are currently being explored for diseases caused by aberrant mRNA splicing. The CRISPR/Cas9 gene-editing technology has successfully corrected mutations in Col4A3 and Col4A5 in foot cells derived from urine (Daga et al., 2020). However, this approach still requires validation in future *in vivo* studies. Exon skipping via antisense oligonucleotides (ASOs) offers therapeutic potential by bypassing faulty splice sites to prevent truncated protein production. This approach has shown promise in treating genetic diseases such as Duchenne muscular dystrophy, hereditary amyotrophic lateral sclerosis, and spinal muscular atrophy (Sang et al., 2024). In Alport syndrome research, ASO therapy has demonstrated efficacy in bypassing specific splicing mutations, improving clinical outcomes in mouse models (Yamamura et al., 2020a). This approach restores COL4A5 chain expression in glomerular and tubular basement membranes, reduces proteinuria, and extends survival in Alport syndrome mice. Additionally, it induces α5 chain expression in iPSC-derived kidney organoids (Yabuuchi et al., 2024). Drug-induced read-through of premature termination codons (PTC) is another promising therapeutic strategy, which has been extensively studied in other genetic diseases such as cystic fibrosis and Duchenne muscular dystrophy (McDonald et al., 2017). Research indicates that AS patients with nonsense mutations are highly sensitive to aminoglycoside-mediated PTC read-through, and some patients may benefit from this treatment (Omachi et al., 2022). ELX-02, a small molecule eukaryotic ribosome-selective glycoside, can induce PTC read-through and is currently undergoing clinical trials (Zheng et al., 2024). Furthermore, the use of molecular chaperones to aid in proper protein folding in the endoplasmic reticulum (ER) can alleviate cell damage caused by ER stress, though this approach has been shown to be most effective for missense mutations, which are associated with milder clinical phenotypes (Hirayama et al., 2023).

In addition to gene therapy, strategies aimed at inhibiting the cell cycle, promoting lipid metabolism and cholesterol efflux in podocytes, and suppressing inflammation and oxidative stress are also considered potential approaches to alleviating Alport syndrome pathology (Zheng et al., 2024). These therapeutic strategies provide new directions for clinical treatment of Alport syndrome, particularly in terms of early intervention for mRNA splicing abnormalities.

This study has several limitations. First, regarding the impact of intronic and exonic SNVs on mRNA splicing, our center lacks synonymous mutations, resulting in an incomplete range of mutation types. Additionally, the small sample size of enrolled patients necessitates further expansion of the cohort to obtain more reliable epidemiological data on the effects of SNVs on mRNA splicing. Second, we employed minigene assay, an *in vitro* method, which only includes exonic regions and part of the flanking introns. For example, the canonical splice site mutation c.2510–2A>G did not result in aberrant splicing in the minigene assay, which may be attributed to the limited genomic fragment included in the construct and the inability of *in vitro* assays to fully recapitulate the endogenous mRNA splicing process. Therefore, the mRNA splicing results obtained through this approach may not always align with *in vivo* findings, and further *in vivo* validation is needed, such as urine mRNA analysis or RNA sequencing of skin fibroblasts (Daga et al., 2018; Wang et al., 2021). Finally, in the study of genotype-phenotype correlations, due to the limited number of patients, we only grouped them into broader categories and did not perform a more detailed analysis of the relationship between different mutation types and clinical phenotypes.

## 5 Conclusion

This study highlights the significant role of mRNA splicing mutations in the COL4A5 gene in Alport syndrome, particularly with 38.5% of SNVs affecting mRNA splicing and leading to more severe clinical phenotypes. This finding offers a new perspective for clinical diagnosis, especially regarding the clinical significance of mutations at non-canonical splice sites. The analysis of the impact of different mutations on renal function emphasizes the importance of early intervention and personalized treatment. Future studies should focus on expanding the sample size and exploring mRNA splicing-based therapeutic strategies, particularly interventions targeting splicing defects through gene therapy, which opens new possibilities for the treatment of Alport syndrome.

## Data Availability

The original contributions presented in the study are publicly available in the LOVD database (User ID: 04100) under the X-linked Alport Syndrome disease entry (ID: 02210) at: https://databases.lovd.nl/shared/diseases/02210.

## References

[B1] Aoto Y. Horinouchi T. Yamamura T. Kondo A. Nagai S. Ishiko S. (2022). Last nucleotide substitutions of COL4A5 exons cause aberrant splicing. Kidney Int. Rep. 7 (1), 108–116. 10.1016/j.ekir.2021.10.012 35005319 PMC8720670

[B2] Boisson M. Arrondel C. Cagnard N. Morinière V. Arkoub Z. A. Saei H. (2023). A wave of deep intronic mutations in X-linked Alport syndrome. Kidney Int. 104 (2), 367–377. 10.1016/j.kint.2023.05.006 37230224

[B3] Canson D. Glubb D. Spurdle A. B. (2020). Variant effect on splicing regulatory elements, branchpoint usage, and pseudoexonization: strategies to enhance bioinformatic prediction using hereditary cancer genes as exemplars. Hum. Mutat. 41 (10), 1705–1721. 10.1002/humu.24074 32623769

[B4] Conesa A. Madrigal P. Tarazona S. Gomez-Cabrero D. Cervera A. McPherson A. (2016). A survey of best practices for RNA-seq data analysis. Genome Biol. 17, 13. 10.1186/s13059-016-0881-8 26813401 PMC4728800

[B5] Daga S. Baldassarri M. Lo Rizzo C. Fallerini C. Imperatore V. Longo I. (2018). Urine-derived podocytes-lineage cells: a promising tool for precision medicine in Alport Syndrome. Hum. Mutat. 39 (2), 302–314. 10.1002/humu.23364 29098738

[B6] Daga S. Donati F. Capitani K. Croci S. Tita R. Giliberti A. (2020). New frontiers to cure Alport syndrome: COL4A3 and COL4A5 gene editing in podocyte-lineage cells. Eur. J. Hum. Genet. 28 (4), 480–490. 10.1038/s41431-019-0537-8 31754267 PMC7080842

[B7] De Silva S. R. Arno G. Robson A. G. Fakin A. Pontikos N. Mohamed M. D. (2021). The X-linked retinopathies: physiological insights, pathogenic mechanisms, phenotypic features and novel therapies. Prog. Retin. Eye Res. 82, 100898. 10.1016/j.preteyeres.2020.100898 32860923

[B8] Di H. Zhang J. Gao E. Zheng C. Huang X. Wang Q. (2022). Dissecting the genotype-phenotype correlation of COL4A5 gene mutation and its response to renin-angiotensin-aldosterone system blockers in Chinese male patients with Alport syndrome. Nephrol. Dial. Transpl. 37 (12), 2487–2495. 10.1093/ndt/gfac002 35020912

[B9] Fu X.-D. Ares M. (2014). Context-dependent control of alternative splicing by RNA-binding proteins. Nat. Rev. Genet. 15 (10), 689–701. 10.1038/nrg3778 25112293 PMC4440546

[B10] Glidden D. T. Buerer J. L. Saueressig C. F. Fairbrother W. G. (2021). Hotspot exons are common targets of splicing perturbations. Nat. Commun. 12 (1), 2756. 10.1038/s41467-021-22780-2 33980843 PMC8115636

[B11] Groopman E. E. Marasa M. Cameron-Christie S. Petrovski S. Aggarwal V. S. Milo-Rasouly H. (2019). Diagnostic utility of exome sequencing for kidney disease. N. Engl. J. Med. 380 (2), 142–151. 10.1056/NEJMoa1806891 30586318 PMC6510541

[B12] Hirayama R. Toyohara K. Watanabe K. Otsuki T. Araoka T. Mae S.-I. (2023). iPSC-derived type IV collagen α5-expressing kidney organoids model Alport syndrome. Commun. Biol. 6 (1), 854. 10.1038/s42003-023-05203-4 37770589 PMC10539496

[B13] Hudson B. G. Tryggvason K. Sundaramoorthy M. Neilson E. G. (2003). Alport’s syndrome, Goodpasture’s syndrome, and type IV collagen. N. Engl. J. Med. 348 (25), 2543–2556. 10.1056/NEJMra022296 12815141

[B14] Jais J. P. Knebelmann B. Giatras I. Marchi M. D. Rizzoni G. Renieri A. (2000). X-linked Alport syndrome: natural history in 195 families and genotype-phenotype correlations in males. J. Am. Soc. Nephrol. JASN 11 (4), 649–657. 10.1681/ASN.V114649 10752524

[B15] Kamura M. Yamamura T. Omachi K. Suico M. A. Nozu K. Kaseda S. (2020). Trimerization and genotype-phenotype correlation of COL4A5 mutants in Alport syndrome. Kidney Int. Rep. 5 (5), 718–726. 10.1016/j.ekir.2020.01.008 32405592 PMC7210609

[B16] Kashtan C. E. (2021). Alport syndrome: achieving early diagnosis and treatment. Am. J. Kidney Dis. 77 (2), 272–279. 10.1053/j.ajkd.2020.03.026 32712016

[B17] Li B. Dewey C. N. (2011). RSEM: accurate transcript quantification from RNA-Seq data with or without a reference genome. BMC Bioinforma. 12, 323. 10.1186/1471-2105-12-323 PMC3163565 21816040

[B18] McDonald C. M. Campbell C. Torricelli R. E. Finkel R. S. Flanigan K. M. Goemans N. (2017). Ataluren in patients with nonsense mutation Duchenne muscular dystrophy (ACT DMD): a multicentre, randomised, double-blind, placebo-controlled, phase 3 trial. Lancet 390 (10101), 1489–1498. 10.1016/S0140-6736(17)31611-2 28728956

[B19] Morinière V. Dahan K. Hilbert P. Lison M. Lebbah S. Topa A. (2014). Improving mutation screening in familial hematuric nephropathies through next generation sequencing. J. Am. Soc. Nephrol. 25 (12), 2740–2751. 10.1681/ASN.2013080912 24854265 PMC4243343

[B20] Nozu K. Nakanishi K. Abe Y. Udagawa T. Okada S. Okamoto T. (2019). A review of clinical characteristics and genetic backgrounds in Alport syndrome. Clin. Exp. Nephrol. 23 (2), 158–168. 10.1007/s10157-018-1629-4 30128941 PMC6510800

[B21] Ohno K. Takeda J. Masuda A. (2018). Rules and tools to predict the splicing effects of exonic and intronic mutations. Wiley Interdiscip. Rev. RNA 9 (1). 10.1002/wrna.1451 28949076

[B22] Olusanya B. O. Neumann K. J. Saunders J. E. (2014). The global burden of disabling hearing impairment: a call to action. Bull. World Health Organ. 92 (5), 367–373. 10.2471/BLT.13.128728 24839326 PMC4007124

[B23] Omachi K. Kai H. Roberge M. Miner J. H. (2022). NanoLuc reporters identify COL4A5 nonsense mutations susceptible to drug-induced stop codon readthrough. iScience 25 (3), 103891. 10.1016/j.isci.2022.103891 35243249 PMC8866893

[B24] Putscher E. Hecker M. Fitzner B. Lorenz P. Zettl U. K. (2021). Principles and practical considerations for the analysis of disease-associated alternative splicing events using the gateway cloning-based minigene vectors pDESTsplice and pSpliceExpress. Int. J. Mol. Sci. 22 (10), 5154. 10.3390/ijms22105154 34068052 PMC8152502

[B25] Richards S. Aziz N. Bale S. Bick D. Das S. Gastier-Foster J. (2015). Standards and guidelines for the interpretation of sequence variants: a joint consensus recommendation of the American College of medical genetics and genomics and the association for molecular pathology. Genet. Med. 17 (5), 405–424. 10.1038/gim.2015.30 25741868 PMC4544753

[B26] Sang A. Zhuo S. Bochanis A. Manautou J. E. Bahal R. Zhong X.-B. (2024). Mechanisms of action of the US food and drug administration-approved antisense oligonucleotide drugs. BioDrugs 38 (4), 511–526. 10.1007/s40259-024-00665-2 38914784 PMC11695194

[B27] Savige J. Ariani F. Mari F. Bruttini M. Renieri A. Gross O. (2019). Expert consensus guidelines for the genetic diagnosis of Alport syndrome. Pediatr. Nephrol. 34 (7), 1175–1189. 10.1007/s00467-018-3985-4 29987460

[B28] Savige J. Lipska-Zietkiewicz B. S. Watson E. Hertz J. M. Deltas C. Mari F. (2022). Guidelines for genetic testing and management of Alport syndrome. Clin. J. Am. Soc. Nephrol. 17 (1), 143–154. 10.2215/CJN.04230321 34930753 PMC8763160

[B29] Savige J. Sheth S. Leys A. Nicholson A. Mack H. G. Colville D. (2015). Ocular features in Alport syndrome: pathogenesis and clinical significance. Clin. J. Am. Soc. Nephrol. 10 (4), 703–709. 10.2215/CJN.10581014 25649157 PMC4386265

[B30] Wahl M. C. Will C. L. Lührmann R. (2009). The spliceosome: design principles of a dynamic RNP machine. Cell. 136 (4), 701–718. 10.1016/j.cell.2009.02.009 19239890

[B31] Wang X. Zhang Y. Ding J. Wang F. (2021). mRNA analysis identifies deep intronic variants causing Alport syndrome and overcomes the problem of negative results of exome sequencing. Sci. Rep. 11 (1), 18097. 10.1038/s41598-021-97414-0 34508137 PMC8433132

[B32] Yabuuchi K. Horinouchi H. Yamamura T. Nozu K. Takasato M. (2024). Investigation of exon skipping therapy in kidney organoids from Alport syndrome patients derived iPSCs. Genes. Cells Devoted Mol. Cell. Mech. 29 (12), 1118–1130. 10.1111/gtc.13170 PMC11609602 39435529

[B33] Yamamura T. Horinouchi T. Adachi T. Terakawa M. Takaoka Y. Omachi K. (2020a). Development of an exon skipping therapy for X-linked Alport syndrome with truncating variants in COL4A5. Nat. Commun. 11 (1), 2777. 10.1038/s41467-020-16605-x 32488001 PMC7265383

[B34] Yamamura T. Horinouchi T. Nagano C. Omori T. Sakakibara N. Aoto Y. (2020b). Genotype-phenotype correlations influence the response to angiotensin-targeting drugs in Japanese patients with male X-linked Alport syndrome. Kidney Int. 98 (6), 1605–1614. 10.1016/j.kint.2020.06.038 32712167

[B35] Zeni L. Mescia F. Toso D. Dordoni C. Mazza C. Savoldi G. (2024). Clinical significance of the cystic phenotype in Alport syndrome. Am. J. Kidney Dis. 84 (3), 320–328.e1. 10.1053/j.ajkd.2024.02.005 38514012

[B36] Zheng Q. Gu X. He J. C. Xie J. (2024). Progress in therapeutic targets on podocyte for Alport syndrome. J. Transl. Intern. Med. 12 (2), 129–133. 10.2478/jtim-2024-0005 PMC11135632 38812923

[B37] Zhou X. Zhou W. Wang C. Wang L. Jin Y. Jia Z. (2020). A comprehensive analysis and splicing characterization of naturally occurring synonymous variants in the ATP7B gene. Front. Genet. 11, 592611. 10.3389/fgene.2020.592611 33719328 PMC7947925

